# Conocimientos, actitudes, prácticas, temor y estrés ante el Covid-19 en estudiantes y recién egresados de Enfermería en Colombia

**DOI:** 10.15649/cuidarte.2044

**Published:** 2021-09-21

**Authors:** Sonia Patricia Carreño-Moreno, Lorena Chaparro-Díaz, Cristian David Cifuentes-Tinjaca, Freddy Elías Perilla-Portilla, Erika Ximena Viancha-Patiño

**Affiliations:** 1 Enfermera, Doctora en Enfermería, Profesora asociada, Universidad Nacional de Colombia - Sede Bogotá - Facultad de Enfermería- Grupo de Investigación Cuidado de Enfermería al paciente crónico - Carrera 30 No.45 - 03, Edif. 228 of. 306, Bogotá, Código Postal: 111321 Colombia. Email: spcarrenom@unal.edu.co Autora de correspondencia. Universidad Nacional de Colombia Universidad Nacional de Colombia Colombia spcarrenom@unal.edu.co; 2 Enfermera, Doctora en Enfermería, Profesora asociada, Universidad Nacional de Colombia - Sede Bogotá - Facultad de Enfermería- Grupo de Investigación Cuidado de Enfermería al paciente crónico - Carrera 30 No.45 - 03, Edif. 228 of. 301, Bogotá, Código Postal: 111321 Colombia. Email: olchaparrod@unal.edu.co Universidad Nacional de Colombia Universidad Nacional de Colombia Colombia olchaparrod@unal.edu.co; 3 Universidad de Los Llanos, Villavicencio, Colombia. Email: cristian.cifuentes@unillanos.edu.co Universidad de los Llanos Universidad de Los Llanos, Villavicencio Colombia cristian.cifuentes@unillanos.edu.co; 4 Universidad Francisco de Paula Santander, Chinacota, Colombia. Email: fperilla1@gmail.com Universidad Francisco de Paula Santander Universidad Francisco de Paula Santander Colombia fperilla1@gmail.com; 5 Universidad Pedagógica y Tecnológica de Colombia, UPTC. Sogamoso, Colombia. Email: erika.viancha24@gmail.com Universidad Pedagógica y Tecnológica de Colombia Universidad Pedagógica y Tecnológica de Colombia Colombia erika.viancha24@gmail.com

**Keywords:** Infecciones por Coronavirus, Miedo, Estrés Psicológico, Conocimientos, Actitudes y Práctica en Salud., Coronavirus Infections, fear, Stress Psychological, Health Knowledge, Attitudes, Practice., Infecções por Coronavirus, Medo, Estresse Psicológico, Conhecimentos, Atitudes e Prática em Saúde.

## Abstract

**Introducción.:**

La pandemia por Covid-19 ha tenido una afectación de la salud emocional del recurso humano en salud, a pesar de contar con conocimientos sobre el virus y su manejo, el personal sanitario entre el que está Enfermería, presenta estrés y temor ante el Covid-19.

**Objetivo.:**

Describir y correlacionar el nivel de temor, estrés, conocimientos, actitudes y prácticas frente al Covid-19 en estudiantes y recién egresados de Enfermería en Colombia.

**Método.:**

Se realizó un estudio observacional de carácter transversal y analítico en Colombia entre octubre y noviembre de 2020. Los instrumentos de temor, estrés y conocimientos, actitudes y prácticas ante el Covid-19 fueron respondidos en línea por 1621 estudiantes y recién egresados de Enfermería.

**Resultados.:**

Se observó una media de temor de 20.69 **±** 6.1 puntos, una media de estrés de 89.15 ± 29.5 puntos y frecuencias relativas superiores al 80% para los conocimientos actitudes y prácticas ante el Covid-19. Correlaciones directas fueron observadas entre los conocimientos y actitudes y a su vez, las actitudes con las prácticas. El temor se correlacionó de forma inversa con los conocimientos y de forma directa con el estrés ante el Covid-19.

**Conclusión.:**

Se observa un adecuado nivel cognitivo en los estudiantes y recién egresados de enfermería, pero una deficiencia emocional frente al Covid-19, en términos de estrés y temor. Es necesario establecer estrategias de soporte en salud mental para esta población clave en el control actual y futuro de situaciones de crisis en salud.

## Abstract (en)

**Introduction.:**

The Covid-19 pandemic has affected the emotional health of human resources in health care, despite having knowledge about the virus and its management, health care personnel, including nurses, have shown stress and fear of Covid-19.

**Objective.:**

To describe and correlate the level of fear, stress, knowledge, attitudes and practices regarding Covid-19 in nursing students and recent graduates in Colombia.

**Method.:**

A cross-sectional and analytical observational study was conducted in Colombia between October and November 2020. The instruments of fear, stress and knowledge, attitudes and practices regarding Covid-19 were answered online by 1621 nursing students and recent graduates.

**Results.:**

A mean fear score of 20.69 ± 6.1 points, a mean stress score of 89.15 ± 29.5 points and relative frequencies above 80% for knowledge, attitudes and practices to Covid-19 were observed. Direct correlations were observed between knowledge and attitudes and, in turn, attitudes with the practice. Fear was inversely correlated with knowledge and directly correlated with Covid-19 stress.

**Conclusion.:**

An adequate cognitive level is observed in nursing students and recent graduates, but an emotional deficiency in the Covid-19, in terms of stress and fear. It is necessary to establish mental health support strategies for this key population in the current and future control of health crisis situations.

## Resumo

**Introdução.:**

A pandemia de Covid-19 teve um impacto sobre a saúde emocional dos trabalhadores da saúde, apesar de terem conhecimento do vírus e seu manejo, os trabalhadores da saúde, incluindo os da Enfermagem, estão estressados e com medo da Covid-19.

**Objetivo.:**

Descrever e correlacionar o nível de medo, estresse, conhecimento, atitudes e práticas em relação à Covid-19 em estudantes de enfermagem e recém-formados na Colômbia.

**Método.:**

Um estudo observacional, transversal e analítico foi realizado na Colômbia entre outubro e novembro de 2020. Os instrumentos de medo, estresse e conhecimento, atitudes e práticas em relação ao Covid-19 foram respondidos online por 1621 estudantes e recém-formados de enfermagem.

**Resultados.:**

Foi observada uma pontuação média de medo de 20,69 ± 6,1 pontos, uma pontuação média de estresse de 89,15 ± 29,5 pontos e frequências relativas acima de 80% para conhecimentos, atitudes e práticas sobre a Covid-19. Foram observadas correlações diretas entre conhecimento e atitudes e, por sua vez, atitudes com práticas. O medo estava inversamente correlacionado com o conhecimento e diretamente correlacionado com o estresse da Covid-19.

**Conclusão.:**

Um nível cognitivo adequado foi observado em estudantes de enfermagem e recém-formados, mas com deficiência emocional em termos de estresse e medo quando confrontados com a Covid-19. É necessário estabelecer estratégias de apoio à saúde mental para esta população-chave na gestão atual e futura de situações de crise sanitária.

## Introducción

La propagación mundial del SARS-CoV-2 y la mortalidad asociada a Covid-19 han mostrado las dimensiones de una pandemia que, en el caso colombiano, al 11 de diciembre de 2020 ha dejado 1.399.911 casos, 38.484 muertes y todo el territorio nacional afectado(1). La historia epidemiológica del Covid-19 en Colombia, ha pasado por la identificación del primer caso el 6 de marzo de 2020, lo cual dio apertura a la fase de contención, con la posterior diseminación de casos a diferentes departamentos y dificultad en el seguimiento epidemiológico e identificación de la fuente de contagio, lo que enfrentó al país a la circulación comunitaria del virus.

Como respuesta a esta contingencia, el gobierno nacional ha desarrollado una serie de medidas entre las que se destacan la emisión de decretos para ordenar el confinamiento en casa, la prevención del contagio, la prohibición de eventos, la preparación del sistema de salud para la atención y la organización de la economía en tiempos de pandemia. Una de las líneas de acción del gobierno, que ha sido objeto de crítica y protesta, es la relacionada con la organización del talento humano en salud, la cual se ha calificado de atropello a los derechos de los trabajadores, por considerar que se les obliga a abordar en primera línea a la pandemia, sin las garantías laborales adecuadas, en términos de salarios, prestaciones sociales, incentivos y elementos de protección personal(2)-(3). Para el 12 de diciembre de 2020, 21.101 trabajadores de la salud fueron infectados, de los cuales 103 fallecieron, siendo el personal más afectado el de medicina y enfermería(4).

Uno de los aspectos que se ha resaltado en medio de los profesionales de salud, entre ellos el personal de enfermería, es el miedo. Existen temores comunes entre enfermería, dentro de los que se destacan el miedo a contagiarse, a llevar el virus en su cuerpo o prendas de vestir y contagiar a sus seres queridos en casa, a que el sistema de salud colapse y finalmente, el miedo a la discriminación. Además del miedo, otros sentimientos como la frustración, la impotencia, ansiedad y la rabia son comunes, pues se enfrentan a una situación aún desconocida en muchos aspectos, no se sienten suficientemente capacitados, se sienten subvalorados y vulnerables(5)-(6). En el caso de estudiantes y recién egresados de enfermería, el temor es una constante, pues sumado las preocupaciones naturales que sienten en su proceso de formación y en particular cuando están cerca titularse, se suma la posibilidad del desborde del sistema de salud en Colombia y la necesidad de salir a laborar en medio de la pandemia tal como lo previó el Gobierno ante la contingencia(7)-(8).

Parkpour y Griffiths (2020), describen el temor como un estado emocional desagradable producido por la percepción de peligro. Debido al temor, algunos individuos han tenido intentos suicidas debido a que pensaban que tenían Covid-19, incluso cuando sus autopsias concluyeron que no lo tenía(9). El temor ha sido estudiando desde diferentes perspectivas psicológicas y situaciones estresantes tales como el cáncer de mama, demencia, enfermedad de Alzheimer, dolor, cirugía, procedimientos odontológicos, hablar en públicos y fobias(9). Por ello valorar el miedo es importante, dado que permite abordarlo y prevenirlo. Resultado de este estudio se diseñó la FCV-19S, una escala que permite valorar el temor ante el Covid-19(9).

Asimismo, los autores señalan que el temor al Covid-19 también es una herramienta para acrecentar los comportamientos preventivos.

Además de ser una percepción nociva, el temor también puede ser un factor predictor de comportamientos positivos. Harper y Cols (2020), reportaron que el temor ante el covid-19 fue el único predictor ante el cambio social positivo, es decir, el distanciamiento social y lavado de manos. En este sentido, se presentó como una implicación importante, no solo en la comprensión de las respuestas comportamentales humanas ante una pandemia, sino en la utilidad de las emociones negativas en el contexto de la prevención, dado que las emociones negativas pueden servir para desarrollar mejores formas adaptativas y protectoras y en ciertos contextos, mantener a las personas a salvo. No obstante, es necesario considerar, que el temor puro, puede evolucionar a formas patológicas, por lo que es necesario valorarlo e intervenirlo(10). En cuanto a estrés ante el Covid-19 se observaron altos niveles de estrés y ansiedad principalmente en médicos y enfermeras, con cuadros severos de síntomas mentales en hasta el 14.5% de los casos. Se observaron como factores asociados a los síntomas mentales, la edad, el sexo, la especialidad y la proximidad con los pacientes(11). Petzold y cols (2020), señalan la importancia de considerar la alta incidencia de estrés y angustia psicológica en profesionales de la salud, mediante el apoyo en la gestión de emociones fuertes, garantía de la satisfacción de necesidades básicas, comunicación clara, distribución de tareas, horarios de trabajo flexibles y prestación de servicios de ayuda psicosocial(12). Los trabajadores de la salud desarrollan estrés psicológico relacionado con sentirse en peligro, la posibilidad de autoinocular el virus, la preocupación por infectar a familiares y la pobre calidad en el sueño(13).

Ante una situación como la actual, estudios de conocimientos, actitudes y prácticas (CAP) son requeridos en profesionales de la salud. En Italia, Moro y Cols., (2020) reportaron una media de respuestas correctas acerca de la epidemia del 71,6% para los trabajadores de salud y del 61,2% para los trabajadores en general. En cuanto al manejo de los pacientes, el 57,8% de los trabajadores acertaron acerca de los tratamientos; sin embargo, se requiere fortalecer las medidas preventivas de contagio y los comportamientos(14). En Henan China, un estudio encontró que el 89% de los trabajadores tenían adecuados conocimientos, más del 80% temían auto inocular el virus y el 89% seguían de manera adecuada las prácticas para contener el virus. Los trabajadores con mayor experiencia, mejor categoría laboral y menos factores de riesgo para covid-19, presentaron mejores actitudes hacia el mismo(15). Por otra parte, un estudio realizado en Irán con estudiantes del área de salud, encontró que estos tenían buenos conocimientos relacionados con covid-19, lo que se evidenció en que más del 85% presentó respuestas acertadas ante las preguntas sobre la temática. Además, los comportamientos preventivos se dieron el más del 94% de los casos y se observó una percepción de riesgo moderado de 4.08/8(16).

Acorde con el panorama presentado, se observa que el temor, estrés, conocimientos, actitudes y prácticas ante el COVID-19 son áreas de interés para estudio en la población en general y aún más para el personal de salud. En la literatura revisada, se pone de manifiesto la importancia de conducir estudios en asuntos relacionados con las emociones, percepciones y comportamientos, pues, aunque la investigación enfocada en la biología del virus propiamente dicha es urgente y fundamental, la investigación en el campo comportamental es prioritaria para lograr un control integral de la propagación del virus y conocer las consecuencias conexas a la situación de pandemia(9).(10).(14). Además, dados que dichos estudios se han conducido en el ámbito internacional, es necesario conducirlos en el contexto colombiano. La hipótesis de este estudio es que existen niveles inadecuados de CAP, temor y estrés ante el COVID-19, además de correlaciones entre estas variables entre estudiantes y recién egresados de enfermería. Así, el objetivo de este estudio es describir y correlacionar el nivel de temor, estrés, conocimientos, actitudes y prácticas frente al Covid-19 en estudiantes y recién egresados de Enfermería en Colombia.

## Materiales y métodos

### Tipo de estudio

Se realizó un estudio observacional de carácter transversal y analítico entre octubre y noviembre de 2020. Este tipo de diseño es adecuado para la descripción de problemas de investigación de los que se tiene poca información previa. Permite describir las variables de interés, dar cuenta de la magnitud del problema e identificar sus factores asociados(17)-(18).

### Participantes

La muestra estuvo constituida por 1621 estudiantes y recién egresados de Enfermería en Colombia que tienen contacto con la Asociación Colombiana Estudiantil de Enfermería - ACOEEN. Se captaron participantes de todo el territorio colombiano mediante un muestreo por conveniencia. Fueron criterios de inclusión: Ser estudiante de último año de enfermería o haber egresado hace máximo un año para el momento del estudio, tener 18 años o más de edad, estudiar y residir en Colombia. Fueron criterios de exclusión: para los estudiantes de enfermería, estar laborando como técnicos de enfermería en alguna institución que cuida persona con COVID-19.

### Instrumentos

Los instrumentos fueron:

*a) Fear of COVID-19 Scale*, la cual es una escala unidimensional de 10 ítems desarrollada por Ahorsu y Cols en 2020. La validez de constructo de esta escala reportó la unidimensionalidad con cargas factoriales superiores a 0.66 y una correlación ítem - total de 0.47 a 0.56. La consistencia interna del instrumento en su versión original es aceptable, con un alfa de Cronbach de 0.82 y una estabilidad aceptable con un coeficiente de correlación intraclase de 0.72. La escala puede ser auto administrada y cuenta con una escala de respuesta tipo Likert de cinco puntos que van desde nunca hasta siempre(19). Para la interpretación del instrumento se debe considerar que no cuenta con puntos de corte y que, a mayor puntaje, mayor temor ante el COVID-19. La puntuación máxima posible es 50 puntos. Para la muestra del presente estudio, se observó un alfa de Cronbach de 0.87.

*b) Knowledge, attitudes, and practices towards COVID-19,* el cual cuenta con 12 preguntas, cuatro relacionadas con la presentación clínica de la enfermedad, tres con los mecanismos de transmisión y cinco correspondientes a la prevención y control. Las preguntas tienen opción politómica con opciones falso, verdadero y no sé. A cada respuesta acertada, se le asigna un valor de 1 punto, por lo que, el cuestionario va desde cero hasta doce puntos. Para efectos de presentar los descriptivos del cuestionario, en la subescala conocimiento se incluyeron las respuestas incorrectas y la categoría no sé en una sola llamada respuesta incorrecta. El cuestionario cuenta con un alfa de Cronbach de 0.71 en su versión original(20), mientras que para la muestra de este estudio se calculó en 0.79. Para la interpretación del instrumento se debe considerar que no cuenta con puntos de corte y que, a mayor puntaje, mayores conocimientos, actitudes y mejores prácticas ante el COVID-19. La puntuación máxima posible es 12 puntos en conocimientos, 2 en actitudes y 2 en prácticas.

*c) COVID Stress Scale,* el cual es un instrumento de 36 ítems con escala de medición tipo Likert de 5 opciones que van desde nunca hasta siempre. El instrumento cuenta con seis dimensiones determinadas a través de un análisis factorial confirmatorio. Las dimensiones son: peligro de covid-19 y contaminación, consecuencias socioeconómicas del covid-19, Xenofobia al covid-19, estrés traumático al covid-19 y comportamiento compulsivo ante el covid-19. La consistencia interna a través del coeficiente alfa de Cronbach, mostró valores superiores a 0.8 en todas las dimensiones, con correlaciones superiores a 0.4 en su versión original(21). Para la interpretación del instrumento se debe considerar que no cuenta con puntos de corte y que, a mayor puntaje, mayor estrés ante el COVID-19. La puntuación máxima posible es 180 puntos. Para la muestra de este estudio se calculó un alfa de Cronbach de 0.84.

### Procedimientos

Los instrumentos de caracterización y de medición de variables de interés fueron distribuidos a los correos electrónicos de los participantes, siendo recolectada la información a través de formularios de Google. El estudio fue aprobado por el Comité de Ética de la Facultad de Enfermería de la Universidad Nacional de Colombia con el registro AVAL-011-20. Se aplicó el consentimiento informado por medio electrónico en encuesta de Google Forms. Dentro del proceso de consentimiento informado se explicaron los objetivos y justificación del estudio; se explicó además que el formulario de Google no colectaría información personal con el fin de garantizar el anonimato y confidencialidad. Este estudio se clasificó en riesgo mínimo y cumplió con las normas para la investigación con seres humanos(22)-(23).

Se obtuvo permiso de los autores para el uso de los instrumentos. Los instrumentos fueron traducidos por un traductor oficial y su lenguaje revisado por un comité conformado por un lingüista y los cinco investigadores.

## Resultados

En este estudio se observó una mayor representación de estudiantes de enfermería con un 80.2% de la muestra, con baja prevalencia de hábito de fumar, apenas un 2.9% y principalmente de la región central de Colombia con un 71.8%; lo que se dio conforme a lo previsto, pues la mayoría de las unidades académicas de enfermería se encuentran ubicadas en la región central del país. La muestra de este estudio reportó un 6.4% de prevalencia de síntomas asociados a Covid-19, el 13,5% ha sido contacto de un caso sospechoso, el 9.8% ha sido contacto de un caso confirmado, el 46.1% convive con población clasificada en riesgo de enfermedad grave por Covid-19 y el 18% convive con trabajadores de salud en servicio activo a personas con Covid-19 (Ver Tabla 1).

**Tabla 1:** Características demográficas de la muestra de este estudio.

Variables Sociodemográficas				Recuento	%
Grado máximo de escolaridad:	Recién egresado	321	19,80
	Estudiante enfermería	733	80,20
Fuma:	No	1574	97,10
	Si	47	2,90
Estrato socio económico:	1	294	18,14
	2	662	40,84
	3	555	34,24
	4	99	6,11
	5	8	0,49
	6	3	0,19
Región de residencia:	Amazonia	19	1,17
	Andina	1164	71,81
	Caribe	223	13,76
	Orinoquia	104	6,42
	Pacífica	111	6,85
Edad	Promedio ± DE (Rango)	20.4 ± 1.84	(18-13)

Dentro de los participantes del estudio, se observó que el 6.35% ha tenido síntomas de sospecha de COVID-19, el 13.51% ha sido contacto de una persona calificada como sospechosa de COVID-19, el 9.8% ha sido contacto de un caso confirmado, el 46% convive con población de riesgo para enfermedad grave y el 17.9% convive con un trabajador de salud activo en asistencia directa. La Tabla 2, presenta los hallazgos mencionados.

**Tabla 2:** Sospecha, contacto y exposición a riesgo de COVID-19 en los participantes

Variable	Respuesta	Recuento	%
Ha tenido síntomas en los últimos 14 días como: tos, dolor de garganta, fiebre, malestar general	Sí	103	6,35
Ha sido contacto de alguna persona clasificada como sospechosa de COVID-19	Sí	219	13,51
Ha sido contacto de alguna persona diagnosticada como infectada con COVID-19	Sí	159	9,81
Vive con alguna población de riesgo ante COVID-19 como: personas con enfermedades crónicas, adultos mayores, niños menores de 1 año	Sí	747	46,08
Alguien de su familia (Convivientes) es trabajador de la salud activo en la asistencia directa	Sí	291	17,95

La Tabla 3, presenta los estadísticos descriptivos de la variable temor ante el Covid-19 total y por dimensiones. Se observó una media de temor de 20.69 puntos (DE 6.1) en una escala máxima posible de 50 puntos.

**Tabla 3:** Descriptivos del temor ante el COVID-19

Escala	M	DE	Mín.	Máx.	IC 95%	
					LI	LS
Total, Temor	20,69	6,109	10	47	20,39	20,99

Nota: M=media, DE= Desviación estándar, Min= valor mínimo, Max= valor máximo, IC95%= Intervalo de confianza al 95%, Li= límite inferior, LS=límite superior.

La Tabla 4, presenta los estadísticos descriptivos de la variable estrés ante el Covid-19 total y por dimensiones. Se observó una media de estrés de 89.15 puntos (DE 29.5) en una escala máxima posible de 180 puntos.

**Tabla 4:** Descriptivos del estrés ante el COVID-19

Escala/Subescalas	M	DE	Mín.	Máx.	IC 95%	
					LI	LS
Total, estrés	89,15	29,589	36	180	87,71	90,59
Peligro	19,82	5,761	6	30	19,54	20,10
Consecuencias socioeconómicas	14,94	6,791	6	30	14,60	15,27
Xenofobia	14,12	6,579	6	30	13,80	14,44
Contaminación	17,29	6,779	6	30	16,96	17,62
Estrés traumático	10,25	5,681	6	30	9,97	10,52
Control compulsivo	12,74	5,737	6	30	12,46	13,02

Nota: M=media, DE= Desviación estándar, Min= valor mínimo, Max= valor máximo, IC95%= Intervalo de confianza al 95%, Li= límite inferior, LS=límite superior.

La Tabla 5, Presenta los estadísticos descriptivos de la variable conocimientos, actitudes y prácticas (CAP) ante el Covid-19, total y por dimensiones. Se observó una media de CAP de 10.4 puntos (DE 2.3) en una escala máxima posible de 14 puntos.

**Tabla 5:** Descriptivos de los conocimientos, actitudes y prácticas ante el COVID-19

Escala/Subescalas	M	DE	Mín.	Máx.	IC 95%	
					LI	LS
Total, CAP	10.4	2.3	6	14	10.12	10.27
Conocimientos	8.32	1.2	4	12	8.03	8.28
Actitudes	1.35	0.32	0	2	1.26	1.29
Prácticas	1.28	0.36	0	2	1.12	1.21

Nota: M=media, DE= Desviación estándar, Min= valor mínimo, Max= valor máximo, IC95%= Intervalo de confianza al 95%, Li= límite inferior, LS=límite superior.

La Tabla 6, presenta las correlaciones significativas entre las variables de estudio. Se observaron correlaciones débiles y negativas entre el temor con las actitudes y las prácticas ante el COVID-19, además, correlaciones débiles y positivas entre el temor con el estrés total y sus dimensiones. Se observaron correlaciones débiles y positivas entre los conocimientos y las actitudes, asimismo, entre las actitudes y las prácticas. Finalmente, se encontraron correlaciones entre moderadas a fuertes y positivas entre el estrés total y sus dimensiones.

**Tabla 6:** Descriptivos de los conocimientos, actitudes y prácticas ante el COVID-19

U	Total Temor	Total conocimientos	Total actitudes	Total prácticas	Peligro	Consecuencias socioeconómicas	Xenofobia	Contaminación	Estrés traumático	Control compulsivo	Total estrés
Total Temor	1										
Total conocimientos	0,003	1									
Total actitudes	-,050*	,070**	1								
Total prácticas	-,056*	0,002	,100**	1							
Peligro	,220**	0,001	0,024	-0,019	1						
Consecuencias socioeconómicas	,166**	-0,042	0,025	0,007	,623**	1					
Xenofobia	,162**	-0,022	0,041	0,006	,513**	,626**	1				
Contaminación	,200**	-0,004	0,015	-0,020	,675**	,522**	,665**	1			
Estrés traumático	,185**	-0,042	-0,001	0,008	,423**	,533**	,554**	,507**	1		
Control compulsivo	,154**	-0,038	0,022	0,002	,433**	,499**	,511**	,500**	,675**	1	
Total estrés	,228**	-0,031	0,027	-0,003	,772**	,809**	,823**	,822**	,767**	,750**	1

Nota: Prueba Correlación de Pearson (r). Valor p: *<0.05 **<0.01 y ***<0.001

## Discusión

Este estudio tuvo como objetivo describir y correlacionar el nivel de temor, estrés, conocimientos, actitudes y prácticas frente al Covid-19 en estudiantes y recién egresados de Enfermería en Colombia. Estas variables, como se observó a lo largo de la revisión de la literatura, son transversales para conocer aspectos cognitivos, emocionales y comportamentales ante el COVID-19 y su conocimiento se convierte en un insumo útil para la toma de decisiones frente al desarrollo de estrategias de mejora en la población de estudiantes y recién egresados de enfermería, de cara a su futura vinculación como talento humano en salud que atenderá a personas con COVID-19, pero además, a la toma de medidas prioritarias que en la actualidad permitan la contención del virus y sus efectos en la población de interés(9).(10).(14). En lo posterior, se discuten los principales hallazgos derivados de este estudio, así como sus aportes y limitaciones.

La variable temor ante el Covid-19 estuvo entre 20.39 y 20.99 puntos con una confianza del 95%, lo que ubica este comportamiento en un punto intermedio de temor, dado un puntaje máximo posible de 50 puntos (Ver Tabla 3). No obstante, el nivel de temor ante el Covid-19 reportado en este estudio es mayor al referido por Winter y cols(24) con una media de 15.6 puntos para la muestra uno y de 18.3 puntos para la muestra 2, en Nueva Zelanda, en el momento en que la alerta transitaba hacia el máximo nivel. Llama la atención la superioridad del nivel de temor en Colombia versus el reportado en Nueva Zelanda, considerando que para el momento en que la muestra de este estudio fue colectada, Colombia empezaba a flexibilizar las medidas restrictivas para prevención de contagio y estaba descendiendo el primer pico de la pandemia. Por otra parte, el nivel de temor en este estudio fue similar al reportado por Sakib y cols(25) en su estudio en Bangladesh en población general, en un momento para el que la región se encontraba en alerta máxima y confinamiento. Un factor condicionante del mayor puntaje de temor podría ser el tener la condición de estudiantes o recién egresados de enfermería, dado que se contempla estar en un mayor riesgo de infectarse y transmitir la infección a las familias(26).

Por su parte, el estrés ante el Covid-19 se presentó entre 87,71 y 90.59 puntos de 180 puntos máximos posibles (Ver Tabla 4). Estos niveles de estrés ante el Covid-19 son mucho mayores que los reportados por Montano y Lacaran(27) en la población general de Filipinas, considerando que los dos estudios se dieron en fases similares de la pandemia para los dos países. En el caso colombiano las dimensiones más afectadas fueron las de peligro y contaminación, mientras que la de menor puntuación fue la de estrés traumático. Estos hallazgos, contrastan con el comportamiento actual de la población colombiana, pues a pesar de preocuparte por el peligro y contaminación, cada vez menos se acogen las medidas de control del riesgo(28). Por su parte, Aslan y cols. en su estudio con estudiantes de enfermería, han mostrado niveles moderados de estrés ante el Covid-19, similares a nuestro estudio, encontrándose mayores niveles de estrés en mujeres y en estudiantes que están en semestres iniciales de la carrer(29). El miedo a ser infectado es un patrón identificado por Savitsky y cols, lo que se relaciona con alta prevalencia de ansiedad, en donde la resiliencia actúa como factor moderador(30). Finalmente, frente al estrés, los hallazgos del presente estudio son similares a los reportados en uno conducido en una muestra de España, en donde se observaron niveles leves a moderados de estrés general en el contexto de la pandemia por COVID-19 y el confinamiento, lo que señala que probablemente no solo exista estrés ante el COVID-19 en la muestra del presente estudio, sino que, a nivel general, también puedan agravarse situaciones de estrés general preexistente e incluso, como lo discuten los autores del estudio conducido en España, puedan predecirse trastornos de estrés postraumático(31).

En este estudio, aunque el nivel de conocimientos sobre el Covid-19 es alto, aún presenta niveles subóptimos en todas las áreas (Ver Tabla 5). Se observó, por ejemplo, que las áreas con menor conocimiento son los síntomas, la evolución de la enfermedad a formas graves, el tipo de tapabocas que puede usar la gente del común y la posibilidad de infección al interactuar con animales salvajes. Estos hallazgos son congruentes con los reportados en China en donde, además, se identificó que los estudiantes con buenos conocimientos y actitudes positivas, desarrollan más y mejores comportamientos preventivos(32). En el mismo sentido, Provenzano y cols. identificaron altos niveles de conocimientos, actitudes y prácticas (CAP) en estudiantes de enfermería en Italia, inmediatamente después de la instauración del aislamiento; en contraste con Colombia, el estudio italiano reportó una asociación positiva entre el avance en la carrera y los CAP(33). Dados los resultados de este estudio, puede concluirse que, a pesar de tener altos niveles de CAP, los estudiantes y recién egresados de enfermería tienen una prevalencia moderada de temor y estrés ante el Covid-19. Estos hallazgos, pueden comprenderse a la luz del proceso de transición por el que pasan los estudiantes, pues a pesar de que se esperan unos CAP adecuados dado su proceso de formación, el tener que enfrentar asuntos como la práctica clínica y el riesgo potencial que ello implica, hace que situaciones como el temor y estrés sean prevalentes(34)-(35).

En cuanto a las relaciones entre variables, se observó una relación negativa y significativa entre el temor con las actitudes y prácticas ante el Covid-19, hallazgo contrario al informado en un estudio europeo en dónde se informaron diferencias culturales en las regiones europeas, pero no correlaciones entre dichas variables(36). También se halló una relación positiva y significativa entre el temor y el estrés en todas sus dimensiones, similar a lo informado por Rodríguez y cols quienes relatan la compleja relación entre el temor y estrés ante el Covid-19 y su predicción de estados de depresión(37); hallazgos que contrastan con los evidenciados en Colombia, en una muestra de médicos generales, en la que, no se hallaron correlaciones entre las variables(38). Finalmente, se encontraron relaciones positivas y significativas entre los conocimientos y las actitudes; y por otra parte, entre las actitudes y las prácticas; lo que robustece la evidencia frente al papel predictor de los conocimientos en las actitudes y prácticas, además de la necesidad de abordar la prevención del contagio por Covid-19 desde lo cognitivo, pero también desde lo comportamental (Ver Tabla 5)(39)-(40).

Comparado con un estudio similar en cuanto a población, fechas de conducción y variables, conducido en México, la muestra del estudio colombiano reportó menores niveles de temor, estrés, con niveles muy similares de conocimientos ante el COVID-19. En el caso de las actitudes y prácticas, estas variables no fueron reportadas en el estudio mexicano. Dicho estudio, por haberse conducido en el contexto latinoamericano, en un país con nivel de ingresos, medidas de contención, acceso a pruebas y respuesta del sistema de salud similares al contexto colombiano, se constituye en el de mayor comparabilidad. En este sentido, es llamativo que, con conocimientos similares, la muestra colombiana presentó menores niveles de temor y estrés ante el COVID-19. En este sentido y considerando que dichas variables no solo deben verse como negativas, sino en muchos casos como un factor protector ante la posibilidad de contagio, es necesario enfatizar en la población colombiana en las medidas de contención del virus(41).

Es destacable de este estudio, la robustez de la muestra en términos de cantidad y representatividad de todas las regiones de Colombia, en concordancia con las unidades académicas de enfermería que existen en el país. Como limitación, los autores expresan la imposibilidad de generalización de resultados directa, dado que el muestreo se hizo por conveniencia; sin embargo, los resultados pueden ser extrapolables a poblaciones que tengan características similares a las del presente estudio, motivo por el que, se reportó una descripción extensa de las características sociodemográficas de la muestra. Frente a su utilidad práctica, puede asegurarse que el presente estudio ya surtió un primer paso con el desarrollo de dos jornadas (de 10 sesiones cada una) de capacitación en COVID-19 abiertas a los miembros de la ACOEEN y personal de enfermería en general, dada que se ha demostrado que fortalecer los conocimientos, impacta directamente en variables como las actitudes, prácticas, estrés, miedo, ansiedad y depresión, pues a partir de ello, se promueven los mecanismos de afrontamiento, los cuales son necesarios y prioritarios ante esta situación que obliga al confinamiento y por tanto conmina a lidiar con emociones negativas relacionadas(42).

## Conclusiones

Los estudiantes y recién egresados de Enfermería de Colombia presentan moderados niveles de temor y estrés ante el Covid-19, además de una correlación directa entre estas dos variables, es decir, a mayor estrés, mayor temor ante el COVID-19. Se observó además un alto nivel de conocimientos, actitudes y prácticas, con una relación directa entre los conocimientos y actitudes, y las actitudes con las prácticas. Se requiere, por tanto, fortalecer el componente cognitivo frente al COVID-19 con el fin de impactar de forma positiva en las actitudes y las prácticas.

Se observa un adecuado nivel cognitivo pero una deficiencia emocional frente al Covid-19, lo que muestra la necesidad de establecer estrategias de soporte y contención emocional para esta población clave en el control actual y futuro de situaciones de crisis en salud. Es pertinente considerar que, aunque bajos niveles de temor y estrés ante al COVID-19 son el escenario ideal, deben prevalecer las estrategias de sensibilización sobre el riesgo para mantener estrategias de contención.
